# Cardiovascular risk factors awareness and prevalence among primary care physicians: an insight from the West region Awareness Initiative Survey to fight cardiovascular disease (WAIT-CVD) in Cameroon

**DOI:** 10.1186/s13104-015-1747-y

**Published:** 2015-12-09

**Authors:** Ahmadou M. Jingi, Jean Jacques N. Noubiap

**Affiliations:** Department of Internal Medicine and Specialties, Faculty of Medicine and Biomedical Sciences, University of Yaoundé I, Yaoundé, Cameroon; Department of Medicine, Groote Schuur Hospital, University of Cape Town, Observatory, Cape Town, 7925 South Africa; Medical Diagnostic Center, Yaoundé, Cameroon

**Keywords:** Cardiovascular risk factors, Primary care physicians, Cameroon, Sub-Saharan Africa

## Abstract

**Background:**

Data on the frequency and awareness of cardiovascular risk factors in practicing doctors are lacking in Cameroon. This study reports on the prevalence of cardiovascular risk factors in primary care physicians (PCPs) at the forefront for the fight against chronic diseases, and the implications for cardiovascular disease prevention and management.

**Methods:**

We carried out a cross-sectional study in the west region of Cameroon. Participants were recruited from 111 PCPs who lived and worked in the region at the time of the study. Data were collected on designed questionnaires adapted from the WHO STEPS approach in two steps, and a nurse-led examination was performed.

**Results:**

Sixty five (65) consenting doctors, aged 39.1 (SD 8.9) years, with 45 (69.2 %) males, were included. Self-reported hypertension rate was 4.6 % (n = 3). The frequency of pre-hypertension was 21.5 % (n = 14) and of hypertension was 26.2 % (n = 17). Self-reported diabetes rate was 3.1 % (n = 2). The frequency of overweight was 46.2 % (n = 30), and obesity was 23.1 % (n = 15). Eight (12.3 %) participants were smokers, 25 (38.5 %) had excessive alcohol consumption (more than two drinks per day for men and one drink per day for women) and 54 (83.1 %) practiced physical exercise, although below the recommendations. Positive family history any CVD was reported in 52.4 % (39.4–65.1). Up to 35.4 % (23.9–48.2) have never done their lipid profile test. There was no difference in cardiovascular risk factors between males and females, except for systolic blood pressure (*p* < 0.001) and diastolic blood pressure (*p* = 0.002) that were higher in males. No significant difference was noted in the other risk profiles and the rate of awareness between sexes.

**Conclusion:**

There are high prevalence of cardiovascular risk factors with low awareness among PCPs in the West region of Cameroon. This is alarming as doctors at the fore front for the fight against cardiovascular diseases are not aware of their own risk profile. There is need for more awareness programs targeting doctors so as to prevent a sick population with sick doctors.

## Background

Cardiovascular disease (CVD) is the leading cause of death globally, and disproportionally affects developing countries [1]. The World Health Organization (WHO) estimates that annual mortality due to CVD will approach 25 million by 2030 worldwide, of which about 80 % will occur in low- and middle-income countries [2]. CVD burden is fuelled by the rising prevalence of cardiovascular risk factors, mostly hypertension, diabetes, obesity, dyslipidemia and tobacco use [2]. In Cameroon, a low-income country in Central Africa with a population of 19,406,100 inhabitants [3], the IDF estimated the nation prevalence of diabetes among adults aged 20–79 years at 4.8 % in 2013 [4]. The prevalence of overweight or obesity among adults aged ≥15 was estimated at 26 % in 2006 [5]. Between 1994 and 2003, the prevalence of hypertension increased by 2- to 5-fold in rural and urban Cameroonian men and women. Age-adjusted prevalence rate of hypertension moved from 24.4 to 37.2 % in men and from 20.1 to 37.5 % in women [6]. In a nationwide survey conducted in 2012, we found an age-standardized prevalence rate of hypertension of 29.7 % among urban adults [7].

Because of their rising burden, hypertension, diabetes, obesity and consequential CVD are increasingly receiving more from the Cameroon government [8]. Since 2001, a number of health policies and strategies on CVD have been formulated and adopted by the National Ministry of Public Health, including the creation of a National Obesity Center and the National Diabetes-Hypertension Control Program which aim to promote equitable access to quality health services in order to reduce the morbidity and mortality from these conditions [9]. Within the past 4 years, we initiated some studies to capture the epidemiology of CVD and its management in the west region of Cameroon [10–14]. Whereas there are increasing information about cardiovascular risk factors in the general population and consequently more population-based interventions, very little is known on the cardiovascular risk profile and health of healthcare workers. This study which is part of the West region Awareness Initiative Survey to fight cardio-vascular disease (WAIT-CVD) reports on the prevalence of cardiovascular risk factors in primary care physicians (PCPs) that are at the forefront for the fight against chronic diseases, and the implications for CVD prevention and management.

## Methods

### Ethical consideration

The present study received approval from regional authorities of the Ministry of Public Health for the West Region, acting as the local Ethics Committee. All participants interviewed in the study provided a written informed consent before their inclusion.

### Study population and setting

This study was part of the WAIT-CVD, a project which aimed at assessing the level and determinants of awareness of CVD and risk factors among the populations of the West region of Cameroon, in order to design preventive strategies against CVD in the region. It was a cross-sectional study carried-out in February 2012. The West Region is one of the 10 administrative regions of the country, which had in 2010 a population of 1,785,285 inhabitant [3]. The region is divided into 20 health districts inside which are registered 530 health facilities both of the public and private sectors. There were 111 PCPs working in the region at the time of the study. We included the 65 PCPs, who were present at their work place when the investigators visited, and who consented to be enrolled in the study.

### Data collection and procedure

Data were collected during an interview using a structured pretested questionnaire adapted from the WHO STEPS approach in two steps [15]. In Step 1, we collected demographic data such as age and sex, as well as information on hypertension and diabetes history, drinking habits and tobacco use, physical exercise and last health checks. In Step 2, we measured the resting blood pressures using standardized procedures with an automated blood pressure measuring device (OMRON^®^ M4). The mean of two measures performed at least 3 min apart was used for all analyses. Height was measured with a calibrated stadiometer to the nearest 0.5 cm, weight in light clothes with a Seca Scale balance to the nearest 0.1 kg. Four nurses trained for this survey collected data. All participants were seen once at their post of duty. Investigators had an average of 04 h journey to reach participants’ posts of duty, covering a distance of 05–110 km from the survey coordination site.

### Definitions

We defined hypertension according to the WHO recommendations as a systolic blood pressure (SBP) ≥140 mmHg and/or diastolic blood pressure (DBP) ≥90 mmHg or a patient on antihypertensive treatment [16]. Self-reported cases of diabetes were defined as a history of fasting blood glucose >1.26 g/l or being on anti-diabetic treatment [17]. Fasting blood glucose levels were not measured during the survey (WHO Step 2). Body mass index (BMI) was calculated as weight in kg/height^2^ in m^2^, and BMI-based body habitus was classified as normal weight (BMI 18.5–24.9), overweight (BMI 25.0–29.9) and obesity (BMI ≥ 30.0) [18]. Physical activity (for adults aged 18–64 years) was defined as at least 150 min of moderate-intensity aerobic physical activity throughout the week or at least 75 min of vigorous-intensity aerobic physical activity throughout the week or an equivalent combination of moderate- and vigorous-intensity activity [19]. We assessed current tobacco use (not former) defined as smoking at least one cigarette per day at the time of the study.

### Statistical methods

Data were entered and analyzed using the Statistical Package for Social Sciences (SPSS) version 20.0 (IBM Corp. Released 2011. IBM SPSS Statistics for Windows, Version 20.0. Armonk, NY IBM Corp.). Results are expressed as count with percentage or mean with standard deviation (SD) as appropriate. The Chi squared test was used for categorical variable comparisons and the Student t tests for comparing quantitative variables. The unadjusted prevalence rate was calculated as: Number of existing cases (counts) divided by the population in question times 100. A *p* value <0.05 was considered statistically significant.

This manuscript was written following STROBE guidelines for the reporting of observational studies [20].

## Results

Of the 65 PCPs out of 111 working in the region at the time of the survey, 45 were males (69.2 %, CI 56.6–80.1). Their ages ranged between 25 and 56 years, with a mean age of 39.1 (SD 8.9); 42 (65.6 % CI 52.7–77.1) of them were aged >35 years. The cardiovascular risk profile of the participants is shown in Table 1. Self-reported diabetes rate was 3.1 % (n = 2). The frequency of hypertension was 26.2 % (n = 17). The awareness rate of hypertension was low in this population. Self-reported rate of hypertension was seen in three (4.6 %) of participants, giving an awareness rate of 17.7 % (3/17). Fifteen (23.1 %) participants were obese, eight (12.3 %) were smokers and 11 (16.9 %) were physically inactive. Only men were smokers. Twenty-five (38.5 %, CI 26.7–51.4) had excessive alcohol consumption (more than two drinks per day for men and one drink per day for women). About one-third of participants were not aware of their lipid profile, and all those who were aware reported not having dyslipidemia. The risk profile according to sex and age is shown in Table 2. Systolic blood pressure (*p* < 0.001) and diastolic blood pressure (*p* = 0.002) that were higher in males. Participants used to consume a median of two (IQR 1.00–2.00) drinks without sex difference (*p* = 0.753), over a median of 1.75 (IQR 0.50–3.00) days per week. The most consumed alcoholic beverages were beer (40 %, CI 24.9–56.7) and wine (22.5 % CI 10.8–38.5). Participants practiced physical exercise in an average of 2.0 (IQR 1.00–3.00) days per week with no difference between males and females (*p* = 0.650) and between people aged less than 35 years and those aged 35 years or more (*p* = 0.815). The most frequent physical exercise were walking (50 % CI 35.5–64.5) and jogging (18 % CI 8.6–34.1). Football and tennis were practiced on an equal basis (10 % CI 3.3–21.8).

**Table 1:** Cardiovascular risk profile

Risk factor	Frequency (N)	Percentage (95 % CI)
Blood pressure		
Normal	34	52.3 (39.5–64.9)
Pre-hypertension	14	21.5 (12.3–33.5)
Hypertension	17	26.2 (16.0–38.5)
Diabetes (self-reported)		
Yes	2	3.1 (0.4–10.7)
No	63	96.9 (85–98.3)
Body habitus		
Normal weight	20	30.8 (19.9–43.4)
Overweight	30	46.2 (33.7–59.0)
Obesity	15	23.1 (13.5–35.2)
Tobacco use		
Yes	8	12.3 (5.5–22.8)
No	57	87.7 (77.2–94.5)
Excessive alcohol consumption^a^		
Yes	40	61.5 (48.6–73.3)
No	25	38.5 (26.7–51.4)
Physical exercise		
Yes	54	83.1 (71.7–91.2)
No	11	16.9 (8.8–28.3)
Dyslipidemia		
Do not know	23	35.4 (23.9–48.2)
No	42	64.6 (51.8–76.1)
Family history (any CVD)		
Yes	33	52.4 (39.4–65.1)
No	30	47.6 (33.4–59.1)

^a^More than two drinks per day for men and one drink per day for women

**Table 2:** Cardiovascular risk profile according to sex and age

	Overall (N = 65)	Sex			Age		
		Male (n = 45)	Female (n = 20)	*p* value	>35 years (n = 42)	<35 years (n = 23)	*p* value
Age	39.1 (8.9)	42.2 (7.4)	31.9 (8.1)	<0.001	–	–	–
Systolic blood pressure (mmHg)	123.7 (12.5)	127.8 (12.1)	114.6 (7.9)	<0.001	127.8 (12.5)	115.2 (7.6)	<0.001
Diastolic blood pressure (mmHg)	79.4 (11.1)	80.4 (11.9)	79.3 (7.9)	0.002	81 (9.2)	76.4 (9.7)	0.177
Body mass index	26.9 (3.3)	27 (2.9)	26.6 (4.2)	0.660	27.6 (3.1)	25.7 (3.4)	0.092
Last blood pressure measurement (months)	1.00 (0.25–2.00)	1.00 (0.25–2.75)	1.00 (0.44–1.25)	0.596	1.00 (0.25–2.00)	1.80 (0.56–2.75)	0.779
Last glycaemia measurement (months)	3.00 (1.00–12.00)	2.50 (0.63–6.00)	6.50 (2.50–12.00)	0.549	3.00 (0.75–7.50)	3.00 (1.13–12.00)	0.532
Number of days/week of alcohol use	1.75 (0.50–3.00)	1.00 (0.50–3.00)	2.00 (0.50–2.75)	1.000	1.00 (0.50–3.00)	2.00 (1.00–2.50)	1.000
Number of units of alcohol/consumption per day	2.00 (1.00–2.00)	2.00 (1.00–2.00)	0.75 (0.20–1.00)	0.753	2.00 (1.00–2.00)	2.00 (0.30–2.50)	0.753
Number of days of physical exercise/week	2.00 (1.00–3.00)	2.00 (1.00–4.00)	1.50 (1.00–2.00)	0.650	2.00 (1.00–4.75)	1.00 (1.00–2.50)	0.815

Data are expressed as mean (standard deviation) or median (interquartile range)

## Discussion

We report findings of the WAIT-CVD in PCPs and the implications for CVD prevention and management. High prevalence of cardiovascular risk factors with low awareness was found. Doctors in this setting also showed poor health habits. This is alarming as doctors at the fore front for the fight against cardiovascular diseases are not aware of their own risk profile. This has serious implications in the fight against CVD.

Reports on the vascular health of practicing PCPs are few or inexistent in sub-Saharan Africa (SSA). The awareness rate of hypertension and probably other cardiovascular risk factors in this population of physicians is lower than that of the general population [21, 22]. One out four physicians is hypertensive, a rate lower than the prevalence of 47.5 % reported by Dzudie et al. in a self-selected population in the Center, Littoral, North-West and West Regions of Cameroon [21], or the prevalence of 29.7 % found by Kingue et al. in a recent nationwide survey of hypertension prevalence in Cameroon [7]. This age-restrained population of practicing PCPs were however younger by at least 5 years. Our data suggests higher rates with age consistent with other reports [21, 23]. The self-reported rate of diabetes is similar to that of the general population in 1997–1998 [24]. This is expected to be higher in a WHO Steps 3 survey where fasting blood glucose measurement or oral glucose tolerance tests are carried out [15]. Fasting blood glucose was less frequently checked suggesting a very high rate of diabetes unawareness. The rate of obesity was higher than that of the general population as reported by Kamadjeu et al. (6.5 % in men and 19.5 % in women) or Kengne et al. (11.1 %) [5, 23], suggesting physical inactivity. The rate of self-reported physical activity (83.1 %) was higher than the 8.5 % found by Kengne et al. in a general population [23], but was considered insufficient as this was carried out for less than 3 days according to recommendations. This is supported by the higher rate of obesity reported in this survey. Alcohol use was lower compared to the general population [23], with a lower prevalence rate and the number of drinks per week. The unadjusted prevalence rate of tobacco use was lower compared to that of the general population (16 % in the study by Kengne et al.), with a male predominance [23]. A significant proportion of PCPs have never measured their serum lipids. No large scale study on hypercholesterolemia has been published to date. A significant proportion had a family history of at least one CVD event. No comparable data exists.

This study has some limitations. The small number of participants does not permit us to generalize our findings to PCPs of other regions. Such WAIT-CVD surveys are needed nationwide. We could not accurately quantify obesity on BMI alone. More so, the true prevalence rate of diabetes in PCPs could not be quantified as in a WHO Steps 3 survey, as the main aim of the survey was to create awareness. Also, it was difficult to get fasting participants as we had to travel long distances to meet them at their work site. Participants were entitled to be seen once. However, this study has some strong implications. We succeeded in creating CVD awareness in the formally registered PCPs in the region. This will probably reduce the risk of having sick doctors treating sick people. This study had some insights to future prevention and management of CVD. Epidemiological studies should be reported as per target group. This, we think, will improve personal involvement and concern for those found in this category of people.

## Conclusion

High prevalence of cardiovascular risk factors with low awareness was found in this group of PCPs in a low-income setting, with a high burden of cardiovascular risk factors. A population of sick doctors catering for a sick population will be sub-optimal in their performance in patient care. This survey suggests that epidemiologic studies on cardiovascular risks be performed and reported as per target groups and not just in the general population. This will probably raise personal concern and avoid evasiveness as concerns cardiovascular disease. There is urgent need for more awareness programs at the national level, targeting doctors so as to prevent a sick population with sick doctors.

## References

[1] Lim SS, Vos T, Flaxman AD, Danaei G, Shibuya K, Adair-Rohani H (2012). A comparative risk assessment of burden of disease and injury attributable to 67 risk factors and risk factor clusters in 21 regions, 1990–2010: a systematic analysis for the global burden of disease study 2010. Lancet.

[2] World Health Organization. Atlas of heart disease and stroke. http://www.who.int/cardiovascular_diseases/resources/atlas/en/. Accessed 8 Dec 2015.

[3] Cameroon’s National Institute of Statistics. Statistics year book 2010. In: Chapter 4: Characteristics of the population; 2011. p. 39–52. www.statistics-cameroon.org/downloads/annuaire2010/chap4.pdf.

[4] International Diabetes Federation, IDF (2013). Diabetes atlas.

[5] Kamadjeu RM, Edwards R, Atanga JS, Kiawi EC, Unwin N, Mbanya JC (2006). Anthropometry measures and prevalence of obesity in the urban adult population of Cameroon: an update from the Cameroon burden of diabetes baseline survey. BMC Public Health.

[6] Feuzeu L, Kengne AP, Balkau B, Awah PK, Mbanya JC (2010). Ten-year change in blood pressure levels and prevalence of hypertension in urban and rural Cameroon. J Epidemiol Community Health.

[7] Kingue S, Ngoe CN, Menanga AP, Jingi AM, Noubiap JJ, Fesuh B, Nouedoui C, Andze G, Muna WF (2015). Prevalence and risk factors of hypertension in urban areas of Cameroon: a nationwide population-based cross-sectional study. J Clin Hypertens.

[8] Echouffo-Tcheugui JB, Kengne AP (2011). Chronic non-communicable diseases in Cameroon—burden, determinants and current policies. Glob Health.

[9] Njamnshi A, Hiag AB, Mbanya JC (2006). From research to policy: the development of a national diabetes programme in Cameroon. Diabetes Voice.

[10] Jingi AM, Noubiap JJ, Kamdem P, Wawo Yonta E, Temfack E, Kouam Kouam C, Kingue S (2013). The spectrum of cardiac disease in the west region of Cameroon: a hospital-based cross-sectional study. Int Arch Med.

[11] Jingi AM, Noubiap JJ, Yonta EW, Kamdem P, Obama JM, Kingue S (2014). A centre for the diagnosis and treatment of tuberculosis (CDT) in a resource-limited setting: a dragnet for patients with heart disease?. Arch Public Health.

[12] Jingi AM, Noubiap JJ, Ewane Onana A, Nansseu JR, Wang B, Kingue S, Kengne AP (2014). Access to diagnostic tests and essential medicines for cardiovascular diseases and diabetes care: cost, availability and affordability in the west region of Cameroon. PLoS One.

[13] Noubiap JJ, Jingi AM, Veigne SW, Onana AE, Yonta EW, Kingue S (2014). Approach to hypertension among primary care physicians in the west region of Cameroon: substantial room for improvement. Cardiovasc Diagn Ther.

[14] Jingi AM, Nansseu JR, Noubiap JJ (2015). Primary care physicians’ practice regarding diabetes mellitus diagnosis, evaluation and management in the west region of Cameroon. BMC Endocr Disord.

[15] Organisation Mondiale de la Santé: Le manuel de surveillance STEPS de l’OMS: L’approach STEPwise des maladies chroniques. Génève: Organisation Mondiale de la Santé; 2005. http://whqlibdoc.who.int/publications/2006/9789242593839_fre.pdf. Accessed 8 Dec 2015.

[16] GuidelinesSub-Committee (1999). 1999 World Health Organization/international society of hypertension. Guidelines for the management of hypertension. J Hypertens.

[17] American Diabetes Association (2011). Standards of medical care in diabetes—2011. Diabetes Care.

[18] WHO (2000). Obesity: preventing and managing the global epidemic. Report of a WHO Consultation. WHO Technical Report Series 894.

[19] World Health Organization: Physical activity and adults: recommended levels of physical activity for adults 18–64 years. http://www.who.int/dietphysicalactivity/factsheet_adults/en/. Accessed 8 Dec 2015.

[20] von Elm E, Altman DG, Egger M, Pocock SJ, Gøtzsche PC, Vandenbroucke JP (2007). The strengthening the reporting of observational studies in epidemiology (STROBE) statement: guidelines for reporting observational studies. Bull World Health Organ.

[21] Dzudie A, Kengne AP, Muna WF, Ba H, Menanga A, Kouam Kouam C (2012). Prevalence, awareness, treatment and control of hypertension in a self-selected sub-Saharan African urban population: a cross-sectional study. BMJ Open.

[22] Kamadjeu RM, Edwards R, Atanga JS, Unwin N, KIawi EC, Mbanya JC (2006). Prevalence, awareness, and management of hypertension in Cameroon: findings of the 2003 Cameroon burden of diabetes baseline survey. J Hum Hypertens.

[23] Kengne AP, Awah KP, Feuzeu L, Mbanya JC (2007). The burden of high blood pressure and related risk factors in Urban sub-Saharan Africa Africa: evidence from Douala in cameroon. Afr Health Sci.

[24] Sobngwi E, Mbanya JC, Unwin NC, Kengne AP, Fezeu L, Minkoulou EM, Aspray TJ, Alberti KG (2002). Physical activity and its relationship with obesity, hypertension, and diabetes in urban and rural Cameroon. Int J Obes Relat Metab Disord.

